# Differences in Endocrine and Cardiac Changes in Mares and Her Fetus before, during, and after Parturition in Horses of Different Size

**DOI:** 10.3390/ani10091577

**Published:** 2020-09-04

**Authors:** Christina Nagel, Maria Melchert, Christine Aurich, Jörg Aurich

**Affiliations:** 1 Graf Lehndorff Institute for Equine Science, Vetmeduni Vienna, 16845 Neustadt (Dosse), Germany; christina.nagel@vetmeduni.ac.at; 2 Artificial Insemination and Embryo Transfer, Department for Small Animals and Horses, Vetmeduni Vienna, 1210 Vienna, Austria; maria.melchert@vetmeduni.ac.at (M.M.); christine.aurich@vetmeduni.ac.at (C.A.); 3 Obstetrics, Gynecology and Andrology, Department for Small Animals and Horses, Vetmeduni Vienna, 1210 Vienna, Austria

**Keywords:** cortisol, heart rate, horse, pregnancy, size, progestins

## Abstract

**Simple Summary:**

Monitoring of the pregnant mare and her fetus is based on hormone analysis and heart rate recordings which may differ among small, medium-size, and full-size horses. Therefore, Shetland (*n* = 6), Haflinger (*n* = 8), and Warmblood pregnancies (*n* = 9) were studied before and at foaling. Foal weight always approximated 10% of mare weight but relative placenta weight was highest in full-size mares. The concentrations of progestins (hormones that maintain pregnancy) and cortisol (a hormone involved in the onset of foaling but also in an animal’s response to stress) was highest in full-size mares. Progestin concentration decreased towards foaling while cortisol concentration increased. Heart rate of mares increased before foaling with the most pronounced increase in small mares. Overall, Shetland mares foaled earlier than larger-size mares. At foaling, atrio-ventricular blocks (physiological omission of heart beats) regularly occurred in full-size mares but only occasionally in medium-size and small mares, reflecting differences in heart efficiency. In conclusion, some differences exist before and at foaling in horses of different size.

**Abstract:**

Equine fetomaternal monitoring is based on endocrine and cardiac parameters which may differ among small, medium-size, and full-size horses. Therefore, Shetland (*n* = 6), Haflinger (*n* = 8), and Warmblood pregnancies (*n* = 9) were studied during late gestation and at foaling. Weight of mares, foals and placenta, plasma progestin and cortisol concentration, heart rate and heart rate variability (HRV) were determined. Foal weight always approximated 10% of mare weight but relative placenta weight was highest in full-size mares (*p* < 0.05). Progestin (*p* < 0.001) and cortisol (*p* < 0.05) concentration was highest in full-size mares. Progestin concentration decreased towards parturition (*p* < 0.001) while cortisol concentration increased (*p* < 0.01). Maternal heart rate increased before foaling with the most pronounced increase in small mares (*p* < 0.001). The HRV increased during foaling and decreased when delivery was completed (*p* < 0.001). Changes were most pronounced in full-size mares (*p* < 0.001). Atrio-ventricular blocks regularly occurred in parturient full-size mares but only occasionally in medium-size and small mares (time *p* < 0.05, time × group *p* < 0.05). This may reflect breed differences in cardiovascular efficiency. Fetal heart rate decreased towards birth (*p* < 0.001) with the most pronounced decrease in full-size horses (*p* < 0.01). Fetal HRV showed no consistent changes before birth but increased when the foal was born (*p* < 0.001), this increase being most pronounced in full-size foals (*p* < 0.05). In conclusion, this study demonstrates both similarities and differences in peripartum endocrine and cardiac changes in horses of different size.

## 1. Introduction

Despite advances in equine obstetrics and perinatal medicine, late fetal mortality, abortion, and the birth of compromised neonates are still major problems in horses [1]. Therefore, pregnancies should be closely monitored [2,3]. Equine fetomaternal monitoring has greatly improved with the advent of ultrasonography [2,4], which has led to a better understanding of fetal well-being and of the antepartum changes in the fetus and mare [2,5]. Due to the size of the mother and her fetus, however, fetomaternal ultrasonography provides less information in large domestic animals than in humans or small companion animals.

A key diagnostic parameter for fetal well-being is heart rate, which can easily be evaluated by fetomaternal electrocardiogram (ECG) recordings. In addition to heart rate, heart rate variability (HRV) reflects the fine-tuning of cardiac regulation by the autonomous nervous system. Changes in the regulatory effects of the autonomous nervous system on the cardiac beat-to-beat interval are interpreted as fetal maturation during gestation. Changes in HRV may also indicate a fetal stress–response to unfavorable conditions [6,7,8]. Fetomaternal electrocardiography can be used for continuous long-term monitoring from day 120 of gestation onwards in the fetus and at any time of pregnancy in the mare [7,9,10,11]. Fetomaternal ECG is not limited by the mare’s size and can easily be performed in both extremely small and very large horses [12].

Research animals in studies on equine pregnancy and foaling are usually full-size horses or occasionally larger pony breeds. The results, however, are often generalized to horses of all sizes. In cattle, endocrine changes in the peripartum period in part even differ among breeds [13,14,15] and horses differ at least as much in size, weight, and type as cattle. To the best of our knowledge, differences in the preparation for foaling, at parturition and in the neonatal phase have not yet been compared among horses varying substantially in size and weight. We hypothesized that the increase in maternal cortisol and decrease in progestin concentration that initiate foaling as well as the parturition-associated changes in heart rate and HRV depend in part on size of the mare and her foal. To evaluate such differences, we studied small (Shetland), medium-size (Haflinger), and full-size (Warmblood) mares and their fetuses and foals from three weeks before parturition through foaling and early neonatal adaptation. Plasma progestin and cortisol concentration, heart rate, HRV, and cardiac arrhythmias were determined.

## 2. Material and Methods

### 2.1. Ethical Approval

The study was approved by the competent authorities for animal experimentation in Austria (Federal Ministry for Science, Research and Economy, license BMWFW-68.205/0172-WF/V/3b/2015 and BMWFW.68-205/0087-WF/V/3b/2016) and Germany (Brandenburg State Ministry for Rural Development, Environment and Agriculture, license 2347-A-5-1-2018).

### 2.2. Animals

A total of 23 late pregnant mares and their foals were included into the study. All mares were healthy on a thorough physical examination before the start of the study. Depending on their size, the animals were divided into the following groups: small (*n* = 6, Shetland pony), medium-size (*n* = 8, Haflinger), and full-size (*n* = 9, Warmblood). All foals were pure-bred, i.e., all Shetland, Haflinger, and Warmblood mares had been bred to Shetland, Haflinger, and Warmblood stallions, respectively. Age of the mares and sex of their foals are listed in Table 1 and weight is given in the Results section. Mean age did not differ among mares of different groups. All mares had uncomplicated singleton pregnancies. During the last four weeks of gestation, mares were housed in single boxes with access to an outdoor paddock in small groups during daytime (Shetland and Haflinger mares at Vetmeduni Vienna, Austria; Warmblood mares at the Brandenburg State Stud in Neustadt/Dosse, Germany). All mares were fed hay and mineral supplements adjusted to their individual requirements and Warmblood mares received additional oats twice daily. Water was available at all times. All animals were healthy throughout the study period. Parturition was supervised, but no obstetrical intervention was needed. All foals were healthy and mature. Eighteen hours after birth, all foals had an IgG concentration in blood > 800 mg/dL (Snap Test, Idexx, Hoofddorp, The Netherlands).

### 2.3. Experimental Design

During the last three weeks before the expected date of parturition until after foaling, blood samples were taken and fetomaternal ECG recordings were made. A fetomaternal ECG was recorded for one hour once daily between 6:00 a.m. and 8:00 a.m. before foaling and at parturition from two hours before to two hours after delivery. Heart rate in foals was recorded continuously for two hours after birth. Blood was collected from one jugular vein and for determination of progestins and cortisol, blood samples obtained on days 10, 8, 5, 4, 3, 2, and 1 before foaling in mares and between 30 and 60 min after foaling from the mare and its foal were analyzed. For analysis of heart rate and HRV in mares and their fetuses, ECG recordings from days 20, 15, 10, 5, 3, and 1 before parturition were used. After parturition, time to passage of fetal membranes and weight of placenta were recorded. Time to first suckling of foals at the mare’s udder was evaluated. On day one after parturition, all mares and neonatal foals were weighed.

### 2.4. Hormone Analysis

Blood was collected into heparinized tubes (Vacuette, Greiner, Kremsmünster, Austria) and samples were centrifuged at 1200× *g* for 10 min, plasma was decanted and frozen at −20 °C until analysis. Plasma cortisol concentration was determined with an enzyme immunoassay (Demeditec Diagnostics, Kiel-Wellsee, Germany) as described previously and validated for equine plasma in our laboratory [16]. The intra-assay and interassay coefficients of variation were 3.9 and 7.2%, respectively, and the minimal detectable concentration was 1.5 ng/mL. Progestin concentration in plasma was determined with an enzyme immunoassay for progesterone (ADI-900-011, Assay Designs, Ann Arbor, MI, USA) validated for equine plasma in our laboratory as described previously [10]. The antiserum cross-reacts 100% with 5α-pregnane-3,20-dione, thus besides progesterone measuring accurately the most important pregnancy-specific equine progestin. According to the manufacturer’s information, cross-reactivity is 3.5% with 17-OH-progesterone and <1% for all other steroids tested. The intra-assay and interassay coefficient of variation was 5.8 and 8.4%, respectively. The minimal detectable concentration of the assay was 5.8 ng/mL.

### 2.5. Heart Rate and Heart Rate Variability

Fetomaternal ECGs were recorded with the Televet 100 device and evaluated with the software version 5.1.1 (Engel Engineering, Heusenstamm, Germany) as described previously [7]. This software version is specifically designed for fetomaternal ECG recordings in horses. The Televet 100 device was attached to a belt, which was fixed around the mare’s thorax. Data were sent automatically to a nearby computer using a Bluetooth connection. The Televet 100 ECG device displayed and stored the combined as well as the individual recordings of the mare and fetus [7]. Cardiac recordings in newborn foals were made with the Polar S 810i system (Polar, Kempele, Finland) as described peviously [10]. In non-exercising horses, cardiac beat-to-beat (RR) intervals obtained by ECG and Polar monitors were strongly correlated (*r* = 0.999) and near-identical [17].

For analysis of heart rate and HRV parameters, the Kubios HRV Software (Biomedical Signal Analysis Group, Department of Applied Physics, University of Kuopio, Kuopio, Finland) was used. From the recorded RR intervals, a five-minute sequence was used for determination of heart rate and the HRV variable SDRR (standard deviation of the RR interval) in mares and fetuses.

Cardiac arrhythmias in parturient mares were determined as described previously [18]. Maternal and fetal ECG recordings were evaluated in a manual page-by-page verification and analysis by an experienced operator. During this procedure, maternal ECGs were checked visually for the presence of arrhythmias. The ECG recordings were subdivided into 15-min intervals, and all arrhythmias were classified and counted per time interval for each mare.

### 2.6. Statistical Analysis

Statistical comparisons were made with the SPSS statistics software (version 25.0; SPSS-IBM, Armonck, NY, USA). Data were tested for normal distribution by the Kolmogorov–Smirnov test. Data for gestation length, weight of foal, mare and placenta, time from parturition to expulsion of fetal membranes, time from birth of the foal to first suckling, cortisol and progestin concentrations (measured only at one time point in mares and neonates after foaling) were not normally distributed and were compared among groups by the Kruskal–Wallis Test with post-hoc comparisons by the Mann–Whitney Test in the case of an overall significant difference. Data for heart rate, HRV, cortisol, and progestins were not normally distributed and log transformed before analysis. Comparisons were made by ANOVA with a general linear model (GLM) for repeated measures with time as within subject factor and group as between subject factor. In case of an overall significant difference among groups, pair-wise post-hoc comparisons were made by a Tukey test. A *p*-value < 0.05 was considered significant. All values given are means ± standard error of mean (SEM).

## 3. Results

Gestation length was shorter in small (Shetland) than in full-size (Warmblood) mares (*p* < 0.05) with medium-size (Haflinger) mares not differing from the other two groups. The time from birth of the foal to expulsion of the fetal membranes was shorter in the group of small versus medium and full-size mares (*p* < 0.05). Time to first suckling of the foal did not differ between groups (Table 2).

**Figure 1:**
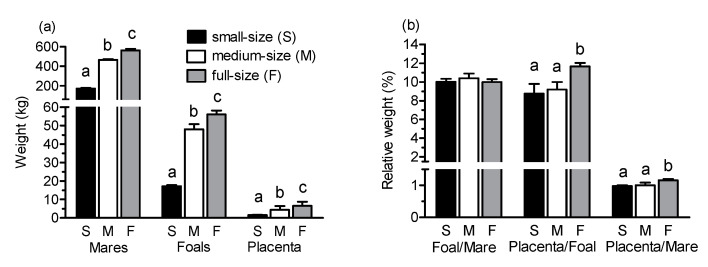
(**a**) Weight of mares, foals and placental weight and (**b**) relative weight (%) foal/mare, placenta/foal and placenta/mare in small (Shetland), medium-size (Haflinger) and full-size (Warmblood) horses (mares and foals on day 1 after parturition). ^abc^ Different letters indicate differences between groups (*p* < 0.05).

The absolute weight of mares and foals on day 1 after parturition as well as placental weight differed significantly among groups (*p* < 0.001; Figure 1a). In all three groups, foal body weight was approximately 10% of mare weight (Figure 1b). In contrast, placental weight in relation to weight of the mares differed among groups with the highest relative placenta weight in full-size mares (*p* < 0.05 vs. both other groups). Foal body weight in relation to weight of the placenta was highest in full-size breed foals (*p* < 0.01) but did not differ between small and medium-size foals.

**Figure 2:**
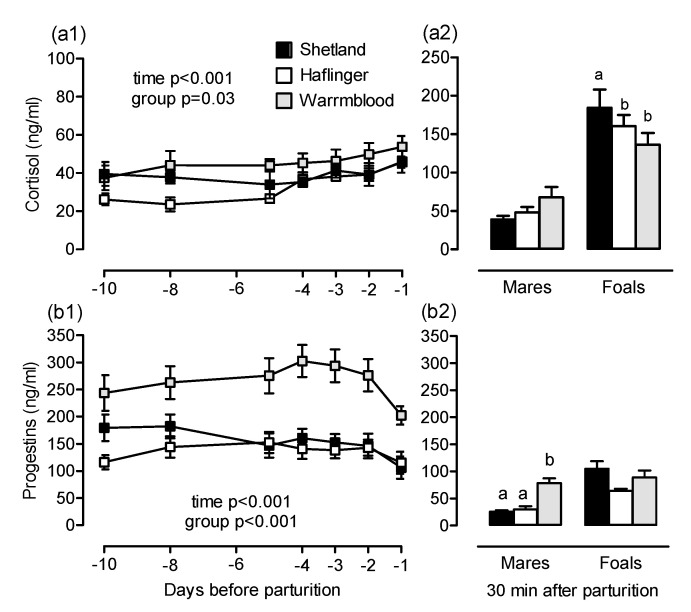
Concentration of (**a**) progestins and (**b**) cortisol in plasma of (**1**) small (Shetland), medium-size (Haflinger) and full-size (Warmblood) mares during the last 10 days before parturition and (**2**) in the same mares and their foals between 30 and 60 min postpartum (always before 1st suckling). Significant differences are indicated in the figures (Figure a2,b2: ^a,b^ different letters indicate significant differences among groups, for pairwise post-hoc comparisons see text). Note different scales on the *y*-axis in Figure a1,a2.

**Figure 3:**
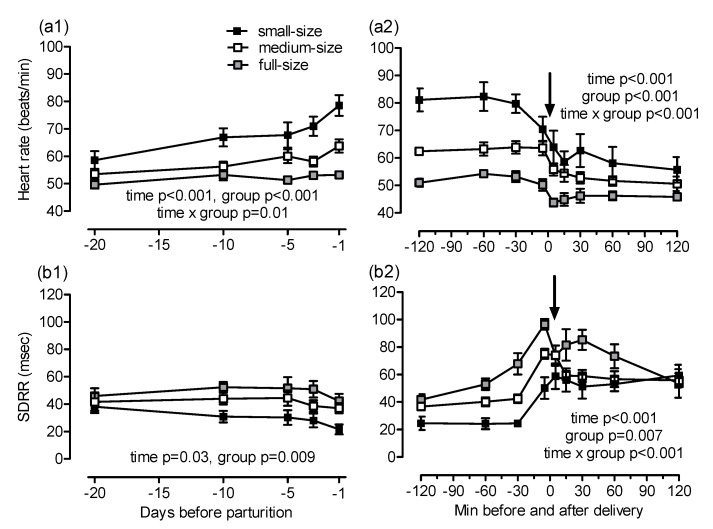
(**a**) heart rate and (**b**) SDRR (standard deviation of the beat-to-beat interval) in small (Shetland), medium-size (Haflinger) and full-size (Warmblood) mares (**1**) during the last 20 days before parturition and (**2**) in the same mares from 120 min before to 120 min after birth of the foal (arrow). Significant differences are indicated in the figures; for pairwise post-hoc comparisons, see text.

Maternal plasma cortisol concentration increased before delivery (*p* < 0.001; Figure 2(a1)). Cortisol concentration differed among groups before foaling and was higher in full-size than in medium-size mares (overall effect *p* = 0.03, post-hoc comparison Warmblood vs. Haflinger *p* = 0.02; Figure 2(a1)) but did not differ after foaling (Figure 2(a2)). Cortisol concentration between 30 and 60 min after birth was higher in foals born to small mares than in foals born to medium or full-size mares (*p* = 0.05; Figure 3(a2)).

At all times, plasma progestin concentration was higher in full-size (Warmblood) mares compared to medium-size (Haflinger) and small (Shetland) mares (overall effect *p* < 0.001, post-hoc comparison Warmblood vs. Haflinger *p* < 0.001, Warmblood vs. Shetland *p* = 0.005; e.g., day −1: Warmblood 202.4 ± 17.0, Haflinger 118.2 ± 22.6, Shetland: 106.62 ± 20.3 ng/mL; Figure 2(b1)). Progestin concentration changed over time in all groups with a decrease during the last days of gestation (*p* < 0.001), but a group difference was still evident after foaling (overall effect *p* = 0.001, full vs. medium-size mares *p* = 0.001, full vs. small-size mares *p* = 0.005; Figure 2(b2)).

**Figure 4:**
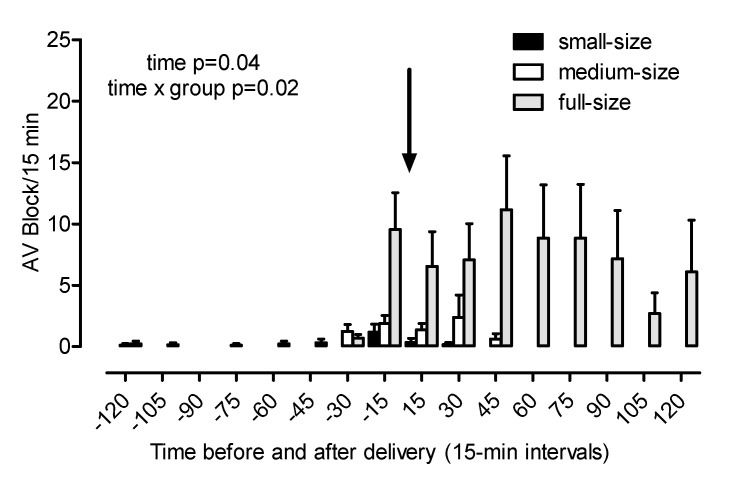
Second degree atrioventricular (AV) blocks (AV blocks per 15 min interval) in small (Shetland), medium-size (Haflinger), and full-size (Warmblood) mares from 120 min before to 120 min after birth of the foal (arrow). Significant differences are indicated in the figure.

Maternal heart rate increased towards foaling (*p* < 0.001; Figure 3(a1)). The earliest and most pronounced increase in heart rate was found in small mares (day −20: 59 ± 3, day −1: 79 ± 4 beats/min), whereas, in full-size mares, the heart rate remained almost constant during the last 20 days of gestation (day −20: 50 ± 1, day −1: 53 ± 1 beats/min; group *p* < 0.001, time × group *p* = 0.015, post-hoc comparisons Warmblood vs. Shetland *p* < 0.001, Warmblood vs. Haflinger *p* = 0.03, Haflinger vs. Shetland *p* = 0.04). Group differences in heart rate were still evident at foaling. In mares from all groups, the heart rate decreased during and shortly after expulsion of the foal and remained largely constant thereafter (time *p* < 0.001, group *p* < 0.001, time × group *p* < 0.001, post-hoc comparison Warmblood vs. Shetland *p* > 0.001, Warmblood vs. Haflinger *p* = 0.02; Figure 3(a2)). Maternal SDRR was nearly identical in all groups on day 20 before foaling and decreased thereafter (time *p* = 0.03). The decrease in HRV was more pronounced in small compared to full-size mares (overall group effect *p* = 0.009, post-hoc comparison Warmblood vs. Shetland *p* = 0.007; Figure 3(b1)). The SDRR increased during the last 30 min of foaling and decreased when delivery of the foal was completed (time *p* < 0.001; Figure 3(b2)). These changes were more pronounced in full-size than in small mares (overall group effect *p* = 0.007, time × group *p* < 0.001, post-hoc comparison Warmblood vs. Shetland *p* = 0.005). Second degree atrioventricular (AV) blocks were the only cardiac arrhythmias observed and were detected only in parturient mares. The AV blocks were most evident in full-size mares from 30 min before to 120 min after delivery. There were less AV blocks in medium-size and small mares and they were observed only until 45 min after complete delivery of the foal (time *p* = 0.04, time × group *p* = 0.02; Figure 4).

**Figure 5:**
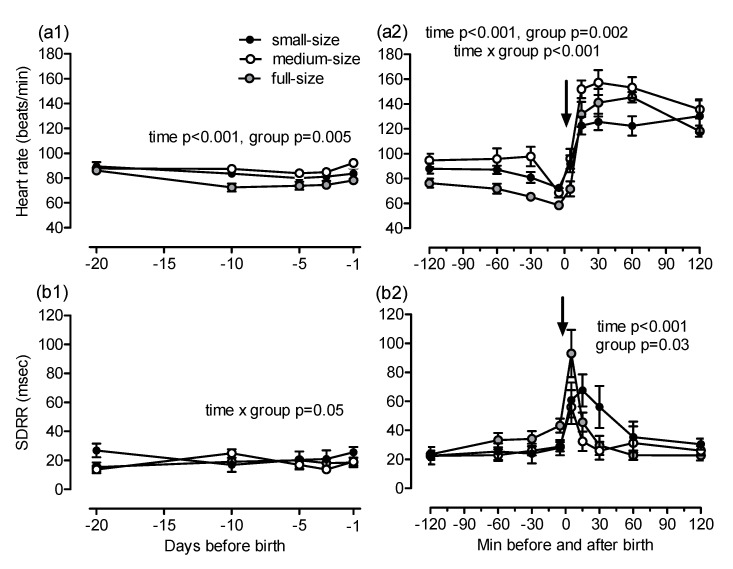
(**a**) Heart rate and (**b**) SDRR (standard deviation of the beat-to-beat interval) in small (Shetland), medium-size (Haflinger) and full-size (Warmblood) fetuses (**1**) during the last 20 days before birth and (**2**) in the same animals from 120 min before to 120 min after birth (arrow). Significant differences are indicated in the figures; for pairwise post-hoc comparisons see text.

Fetal heart rate was close to identical in all groups on day 20 before birth. Fetal heart rate decreased and increased thereafter in all groups (time *p* < 0.001) and differed slightly between full-size and medium-size horses (overall effect *p* = 0.005, post-hoc comparison Warmblood vs. Haflinger *p* = 0.004; Figure 5(a1)). Fetal heart rate decreased during birth and increased markedly in the newborn foal (time *p* < 0.001, group *p* = 0.002, time × group *p* < 0.001, post-hoc comparison Warmblood vs. Haflinger *p* = 0.001, Warmblood vs. Shetland *p* = 0.05; Figure 5(a2)). Fetal SDRR showed no consistent changes over time and among groups before birth (time × group *p* = 0.05) but increased transiently when the foal was born (time *p* < 0.001, group *p* = 0.03, post-hoc comparison Haflinger vs. Shetland *p* = 0.05; Figure 5(b1,b2).

## 4. Discussion

This study demonstrates both similarities and clear differences in characteristic endocrine and cardiac changes before, during, and after parturition in horses of different size. With the Shetland as one of the smallest horse breeds, the Haflinger as a medium-size draft-type breed and the Warmblood sport horse as a large full-size breed, the spectrum of weight and size that exists in horses was well represented in the present study. Because of the number of animals included into the study, our results primarily demonstrate the existence of size differences with regard to the parameters determined and indicate the direction of such differences while not yet allowing to define normal ranges for horses of different size or breed (Table 3).

As expected from the experimental design, the weight of mares, their newborn foals, and the placenta clearly differed among small, medium-size, and full-size horses. In agreement with previous studies [19,20], in all three groups, foal weight was approximately 10% of body weight of their dams. Maternal weight and foal birth weight are positively correlated with the mass and gross area of the allantochorion [20,21,22]. In the present study, relative placental weight did not differ between small (Shetland) and medium-size horses (Haflinger) but was higher in the full-size horses (Warmblood). In addition, foal birth weight in relation to placental weight was highest in the full-size breed foals and did not differ between foals of small and medium-size mares. The findings are in agreement with results from a previous study [22]. Because pony mares have a smaller placenta in relation to their size and body weight than mares of larger horse breeds, there may have been an evolutionary need to enhance placental efficiency by increased development of microcotyledonary blood vessels in order to ensure the birth of mature foals [22]. Small mares released their placenta earlier after birth than medium and full-size mares. While this is of some interest for clinical veterinary medicine, it does not necessarily indicate differences with regard to retained placenta between mares differing markedly in size. The incidence of retained placenta has been reported to differ among breeds, however, and is particularly high in draft horses [23] and in mares of the Friesian breed [24]. The incidence of the retained placenta in horses thus may be positively correlated with mare size or body mass.

Late equine gestation is maintained by progestins originating from the fetoplacental unit [25]. In agreement with a higher relative placental weight in full-size compared to medium-size and small mares, plasma progestin concentrations were also the highest in pregnancies of full-size horses. Although placental efficiency may be to some extent higher in pony breeds [22], progestin synthesis and release appear to depend more on placental size. Compared to mares, progestin concentration was elevated in foals between 30 and 60 min after birth but has been reported previously to decrease markedly during the first hours of life [26].

Second degree AV blocks regularly occurred in healthy full-size Warmblood mares during the expulsive phase of labor and the first two hours after delivery but were only occasionally seen in medium-size Haflinger and small Shetland mares. While AV blocks in parturient Warmblood mares of the present study confirm a previous report [18], their near-absence in parturient Haflinger and Shetland mares is a novel finding. Both full-size Warmblood [18] and small Shetland mares [8] give birth under pronounced parasympathetic dominance. The increase in maternal HRV during and after expulsion of the foal confirms a strong parasympathetic tone at physiological foaling in mares of all groups of this study irrespective of their size. We suggest that the presence and absence of AV blocks at foaling are not a size effect but an effect of horse breed. Because of their highly efficient cardiovascular and respiratory system, fit athletic horses at rest are under a strong parasympathetic tone, resulting in a reduced heart rate and occurrence of AV blocks [27]. Warmblood horses are bred for performance in equestrian sports and thus have a highly efficient cardiovascular system that is to a certain extent comparable to Thoroughbred race horses. Late pregnancy represents a substantial burden for the maternal cardiovascular system in horses [28] and mares towards the end of pregnancy are well adapted to these demands. The increased parasympathetic tone at foaling is associated with frequent AV blocks in Warmblood mares, but no or only individual AV blocks in Haflinger and Shetland mares, i.e., horses with a less efficient cardiovascular system. Cardiac pathologies can be excluded as the reason for AV blocks in our study based both on previous reports [18] and because AV blocks occurred only during and shortly after expulsion of the foal but not at any other time in our study. A breed difference with regard to AV blocks in horses at rest has been reported previously with less AV blocks in small breed horses and in heavy draft horses compared to Thoroughbreds, Warmblood sport horses, and Quarter horses [29].

Fetal adrenocortical activation [30] but also maternal stress associated with increased cortisol release [31,32] initiate the endocrine cascade that finally leads to foaling. Shetland mares in our study foaled earlier than Warmblood mares A shorter gestation length in ponies compared to large-size horse breeds confirms previous studies [21,33,34] but so far has not been associated with fetomaternal stress. It might be argued that the Shetland pony per se may have a shorter gestation than larger horse breeds. In this case, however, one would expect all signs of impending parturition to occur earlier in Shetland mares compared to larger horse breeds which was not the case in our study.

Fetal heart rate was close to identical in all groups 20 days before foaling. Only during the last 20 days of gestation did the fetal heart rate diverge among groups and remained lower in full-size than in medium-size and small mares until foaling. Towards the end of gestation, the fetal autonomous nervous system undergoes maturational changes and this is reflected by a decrease in fetal heart rate and an increase in HRV in all groups. Heart rate usually increases with decreasing size of the animal and thus is higher in postnatal or adult ponies than in large breed horses [29,35]. As parturition approaches, size-related differences in heart rates are thus similar in the equine fetus and the postnatal horse, apparently reflecting maturity of the underlying regulatory mechanisms.

Horses in our study were kept at two different localizations but housing at both places was close to identical with regard to stables, paddocks, and space available per horse. All horses were managed and strictly controlled by our research group. Warmblood horses but not Haflinger and Shetland mares were fed oats in addition to hay, but this difference was due to different energy requirements and would have also occurred if all horsed had been kept in the same place. Comparisons between different types, sizes, or breeds in a given species are usually based on different studies often performed at different times and by different authors utilizing different analytical procedures. In contrast, in the present study, all experimental groups were submitted to an identical sampling and observation protocol and all laboratory parameters were determined together at the same place and with the same assays. Results from the three mare groups studied are therefore directly comparable.

## 5. Conclusions

Although the physiological changes preceding foaling in principal are similar among horses of different size and type, they differ in detail. Foaling occurs under parasympathetic dominance in all horses, but this parasympathetic tone is associated with frequent AV blocks at parturition only in horse breeds selected for athletic performance. Placental progestin synthesis is more efficient in full-size compared to medium size and small mares. These size-related differences have to be taken into account when fetal and maternal well-being needs to be evaluated.

## Figures and Tables

**Table 1:** Age of mares and sex distribution of foals.

	Small (Shetland)	Medium-Size (Haflinger)	Full-Size (Warmblood)
Age (years)	10.8 ± 2.6	9.3 ± 1.2	8.5 ± 1.8
Foal sex (female/male)	0/6	4/4	3/6

**Table 2:** Gestation length, time from birth of the foal to expulsion of fetal membranes and time to first suckling the mare’s udder in small, medium-size and full-size mares and their newborn foals.

	Small (Shetland)	Medium-Size (Haflinger)	Full-Size (Warmblood)
Gestation length (days) (range)	329.5 ± 3.7 ^a^ (312−338)	337.1 ± 2.1 ^ab^ (329−346)	342.8 ± 2.4 ^b^ (333–356)
Expulsion of fetal membranes (min)	20.2 ± 6.0 ^a^	66.7 ± 22.2 ^b^	50.9 ± 8.4 ^b^
First suckling of the foal (min)	136.9 ± 14.5	114.1 ± 10.8	124.7 ± 9.9

^ab^ Different superscript letters indicate differences between groups (*p* < 0.05).

**Table 3:** Suggested mean values for clinical parameters used for assessment of the late pregnant mare, her fetus and newborn foal in horse breeds of different size (note that values are approximate estimates based on a limited number of animals).

	Small (Shetland)	Medium-Size (Haflinger)	Full-Size (Warmblood)
Average gestation length (days)	330 d	340 d	342 d
Release of placenta (min)	≤30 min	≤60 min	≤60 min
Average maternal progestin concentration one day before foaling (ng/mL)	100 ng/ml	100 ng/ml	200 ng/ml
Average maternal heart rate one day before foaling (beats/min)	70 beats/min	60 beats/min	50 beats/min
Average fetal heart rate one day before birth (beats/min)	80 beats/min	80 beats/min	80 beats/min
Neonatal adaptation (time to first suckling, min)	≤2 h	≤2 h	≤2 h
